# Acceptance of a flipped classroom to improve university students’ learning: An empirical study on the TAM model and the unified theory of acceptance and use of technology (UTAUT)

**DOI:** 10.1016/j.heliyon.2022.e12529

**Published:** 2022-12-22

**Authors:** Ibrahim Youssef Alyoussef

**Affiliations:** Faculty of Education, Education Technology Department, King Faisal University, Al Ahsa 31982, Saudi Arabia

**Keywords:** E-Learning, Flipped classrooms, Peer influence, Performance expectancy, Perceived ease of use, Perceived anxiety, Relative advantage

## Abstract

Higher education has given the flipped classroom a lot of attention as a result of its pedagogical success. As a result of the adoption of social media, smartphones, and computers in the classroom, new strategies for providing online courses, such as flipped classrooms, have evolved. To further understand the effects of such technology integration in teaching, the study looked at the responses of 213 undergraduate students at King Faisal University. For a semester, participants took their regular classes in a flipped classroom. The participants answered a survey made expressly for this study to find out if they would still be willing to use flipped classes following this experience. A structural equation modeling approach was used to analyze the research paradigm, which is based on the technological adoption model. The findings demonstrated that each variable category had a favorable influence on perceived usefulness and perceived ease of use. Perceived utility and ease of use serve as mediating elements in the relationship between independent variables and attitudes toward adopting flipped classrooms. Additionally, the findings indicated that ATFC and BIFC have a positive influence on the acceptance of flipped classrooms. In terms of education and learning, the utilization of classroom instruction is positively impacted by both ATFC and BIFC. These findings show that attitudes toward blended learning and intentions to use flipped classrooms have the biggest impacts on the adoption of the concept in Saudi Arabia higher education.

## 1 Introduction

Developing technologies like social media, mobile phones, and tablets have been integrated into the educational system, which has led to the emergence of contemporary web course delivery techniques that aim to improve teaching and learning. A recent blended learning method called the "flipped classroom" assigns asynchronous practice problems and instructional videos as homework while group problem-solving is done in class (Aghaei et al., 2020; Gündüz and Akkoyunlu, 2019; Yang and Chen, 2020). Video lectures are becoming one of the most important resources for flipped classroom implementation (Ahmed and Indurkhya, 2020; Sohrabi and Iraj, 2016). Lectures on demonstrated behavior are produced by teachers and posted to a personal learning management system (LMS) or a public streaming video service (i.e., YouTube, Vimeo, Google Meet) (Al-Emran and Teo, 2020; Al-Rahmi et al., 2019; Chiu and Cheng, 2017; Comber and Brady-Van den Bos, 2018). The class period includes problem-solving, in-depth debates, deeper conceptual study, and peer interaction (Al-Rahmi et al., 2022b; AlJarrah et al., 2018; Nja et al., 2022). Therefore, flipped learning can promote effective teamwork while also increasing critical thinking skills and student satisfaction (Sun and Xie, 2020) (Rawas et al., 2020). The authors also indicate that flipped learning improves student engagement and academic progress (Alamri, 2019; Al-Rahmi et al., 2022a; Gómez-García et al., 2020). The flipped classroom paradigm can assist students in developing their technology and digital literacy as well as their capacity for 21st-century learning (Palazón-Herrera and Soria-Vlchez, 2021; Zainuddin et al., 2019). Thus, delivering a recorded lecture video alongside an advanced faculty member and students can give students an overall, in-depth understanding of the lecture topic. Additionally, it enables individuals to recognize, comprehend, and retain the important ideas of the presentation. A crucial step in making learning relevant is connecting prior knowledge with new information, which is what advanced faculty members and students can do as instructional tools (Palazón-Herrera and Soria-Vlchez, 2021). It may be argued that instructors have recently promoted the use of flipped classrooms by giving their students more opportunities for active learning because these courses play a part in encouraging engagement in interactive and higher-order activities (Wang, 2021). Numerous different studies have been conducted in this field. A few of the advantages mentioned by proponents of flipped classrooms include the strengthening of teacher-student relationships, perceived pleasure, relative advantage, and learning enhancement via active learning in the classroom (Doğan et al., 2021). Other studies discuss the advantages of flipped classrooms, including better student performance, better results, active learning, the usage of flipped classrooms, and higher-order thinking skills (Abdullah Moafa et al., 2018; Bouwmeester et al., 2019). Another category of studies (Day, 2018; Lewis et al., 2018) investigated the efficacy of using flipped classrooms in conjunction with a particular flipped model learning technique. Additionally, the "Research Proposal" course, which is a must for students in the home economics department, primarily depends on their ability to operationalize, identify, and control variables, formulate hypotheses, conduct experiments, and interpret data. Thus, its main goal was to help students develop their skills in the integrated science process in order to meet their needs (Alamri, 2019). However, numerous studies (Alamri, 2019; Doğan et al., 2021; Lewis et al., 2018) in the field of research techniques and procedures have demonstrated that students have issues with such abilities. According to various studies, active teaching and learning techniques are crucial for student learning in higher education. According to (Al-Zahrani, 2015; Alamri, 2019; AlJaser, 2017), the flipped classroom improved students' ability to think critically and creatively in the Saudi educational system. Researchers from the Faculty of Education at King Abdulaziz University in Jeddah, Saudi Arabia, found that the flipped classroom did encourage students' creativity in terms of fluency in coming up with several solutions to a given assignment, flexibility in reaching beyond the known, and uniqueness of ideas. Students also thought that the flipped classroom played a big part in helping them be more creative. This novel technique improves healthcare professions students' school achievement (AlJaser, 2017; Alsaleh, 2020; Evans et al., 2019; Fauzi, 2022). In this model, students can improve their learning by actively collaborating with their peers (Al-Rahmi et al., 2020). Flipping classrooms encourages students to participate in high-quality active learning (Phillips and O'Flaherty, 2019). According to (Núñez et al., 2020), the results reveal that flipped learning is effective both in primary education and in secondary education, being more influential in student development in this last stage. It is concluded that the flipped learning approach has meant an improvement of the academic indicators evaluated after a diet education program. The authors also indicate that 758 teachers, less than half the teachers surveyed, show competences to adequately develop a methodology based on flipped learning, where age, use of information and communication technologies (ICTs) in education, time spent using them in the personal sphere, number of devices and teaching experience have an influence on the application of the method (Moreno-Guerrero et al., 2021).

Furthermore, research demonstrates that adopting flipped classrooms gives teachers greater flexibility and freedom to construct learning techniques and improves students' rational analysis and problem-solving abilities (Phillips and O'Flaherty, 2019). This strategy can help pupils become more motivated if it is implemented correctly (Parra-González et al., 2021). According to Jian (2019), this technique has a considerable impact on effective learning achievements. Despite the fact that the flipped classroom model has gained a lot of popularity due to its benefits (AlJarrah et al., 2018), the adoption rate by learners and the discovery of determining elements that influence students' perceptions of flipped classroom adoption should not be overlooked (Caligaris et al., 2016). In addition to TAM, our study employs the UTAUT theory, which links perceived usefulness, perceived ease of use, attitude toward using Flipped Classroom, and behavioral intention to use Flipped Classroom on academic students in order to promote Flipped Classroom adoption in education. Both UTAUT and the TAM Model are used for the measurement of the adoption of flipping the classroom in education, which has yet to be touched by several studies in the context of Saudi Arabia. As a result, the purpose of this study was to develop a model to identify the key features that are expected to play an important role in students' perceived usefulness and perceived ease of use for learning in their attitudes toward using Flipped Classroom and their behavioral intention to use Flipped Classroom in order to increase their adoption of Flipped Classroom in higher education. To address these issues, this study was conducted to integrate the TAM and UTAUT factors that may affect students' adoption of Flipped Classroom. This study therefore aims to investigate the impact of peer influence (PI), relative advantage (ADV), perceived anxiety (PA), perceived enjoyment (PE), performance expectancy (PEX), effort expectancy (EEX), facilitating conditions (FC) through perceived usefulness (PU), perceived ease of use (PEOU), attitude towards using Flipped Classroom (ATFC), and behavioral intention to use Flipped Classroom (BIFC) on academic students for the adoption of Flipped Classroom (AFC). Thus, the research question was: What are the elements that influence the adoption of flipped classes in Saudi higher education?

### 1.1 Statement of the problem

No unique, comprehensive definition of "flipped classrooms" exists (Abdellah Ibrahim Mohammed Elfeky, 2019; Street et al., 2015). The flipped classroom (FC) may face a number of external challenges, according to existing literature (Betihavas et al., 2016; O'Flaherty and Phillips, 2015). Its successful implementation will depend on the students' and instructors' pedagogical, design, and evaluation skills, claim some academics (Herreid et al., 2014; O'Flaherty and Phillips, 2015; Simonson, 2017). The early results of flipping will influence students' decisions to continue or stop flipping, and they will play a significant role in deciding the FC's continued use (Abeysekera and Dawson, 2015; DeLozier and Rhodes, 2017; Guo, 2019; Sayaf et al., 2021; Simonson, 2017; Xu et al., 2021). However, there is a lack of study on the experiences of instructors and students who have flipped classrooms, as well as their viewpoints on its implementation, advantages, and challenges (Hermanns et al., 2015; Long et al., 2017). Some academics advise enhancing students' understanding of this technique since they think that the deployment of the flipped classroom has been mostly driven by students' intuitive views rather than by scientifically supported principles (Lo and Hew, 2017). Few studies have focused on the requirements of this stage, so (Bernard, 2015) emphasized the need for further investigation of the experiences or concerns of students when making the transition to flipping in his review of the use of the flipped classroom in a variety of higher education disciplines and settings (Simonson, 2017). This study fills a research gap by concentrating on students' opinions of the flipped classroom during the transitional phase (Bernard, 2015). In a Saudi higher education setting, the research will examine the extent to which students and lecturers are aware of the potential of this active-learning strategy for encouraging student-centered learning and encouraging collaborative interaction that strengthens knowledge production. To the best of the researchers' knowledge, the Kingdom of Saudi Arabia has not made any attempts to look into how pupils perceive the FC. By determining the extent to which students have complied with the key requirements of flipping, which will primarily be evaluated in terms of flipped classroom prerequisites, this study will provide documentation on this evolutionary stage (Simonson, 2017). By examining higher education students' early opinions of the flipped classroom, particularly when making their first attempts at flipping, this study aimed to close this research gap. More precisely, this study will reveal how the students view the fundamental requirements and pedagogy of the flipped classroom. It will investigate the motivation for the students' use of it, their judgments of its demands and underlying pedagogy, as well as their perspectives on its advantages and difficulties. It may be possible to secure better results and continuing use in the future by understanding the students' impressions of this stage and the difficulties they confront.

### 1.2 Flipped classrooms

Most Saudi educational institutions have incorporated flipped learning into their pedagogy to help students reach their full potential and in accordance with the Saudi Arabia Education Blueprint (Bakheet and Gravell, 2020). The flipped classroom is a methodical way for teachers to incorporate technology into the classroom (Al-Zahrani, 2015). (The design, execution, and evaluation of a flipped (inverted) classroom approach) for a course on sustainable education The term "flipped classroom" refers to a recently developed teaching method that is frequently used with undergraduate students. The fundamental goal of this instructional strategy is to speed up the acquisition of novel ideas and cutting-edge techniques through the use of pedagogical techniques appropriate for a university setting (Yanbing, 2019). According to (Gómez-García et al., 2020a, Gómez-García et al., 2020b) The findings obtained showed that the application of these methods promoted an increase in students’ motivation, as well as in their autonomy and self-regulation when facing the contents of the subject. For this reason, it is advocated that there is a need to continue promoting a quality and innovative educational practice according to the figure of the student today. We can say that current technology, such as e-learning management systems, may be leveraged to provide pre-prepared courses when contemplating the flipped classroom as a teaching strategy. This makes it possible for students to receive video and audio lectures outside of the classroom via their smartphones, tablets, or laptops, allowing self-directed learning. Nevertheless, self-directed education in flipped classrooms necessitates that students be well-prepared before beginning lectures or classes (Barrie et al., 2018). Therefore, a flipped classroom methodology has been widely adopted by educators of all levels and across all disciplines, and it may be considered a good alternative to not only the traditional classroom or presentation but also to the bulk of conventional teaching techniques (Steen-Utheim and Foldnes, 2018). By more effectively allocating instructional time, it has the fascinating potential to promote students' active learning, cooperation, and mentorship throughout the learning process (Sergis et al., 2018). Advanced organizers make face-to-face sessions more student-centered by allocating time for discussion, group discussions, and problem-solving (Palazón-Herrera and Soria-Vlchez, 2021).

## 2 Theoretical framework

**Figure 1:**
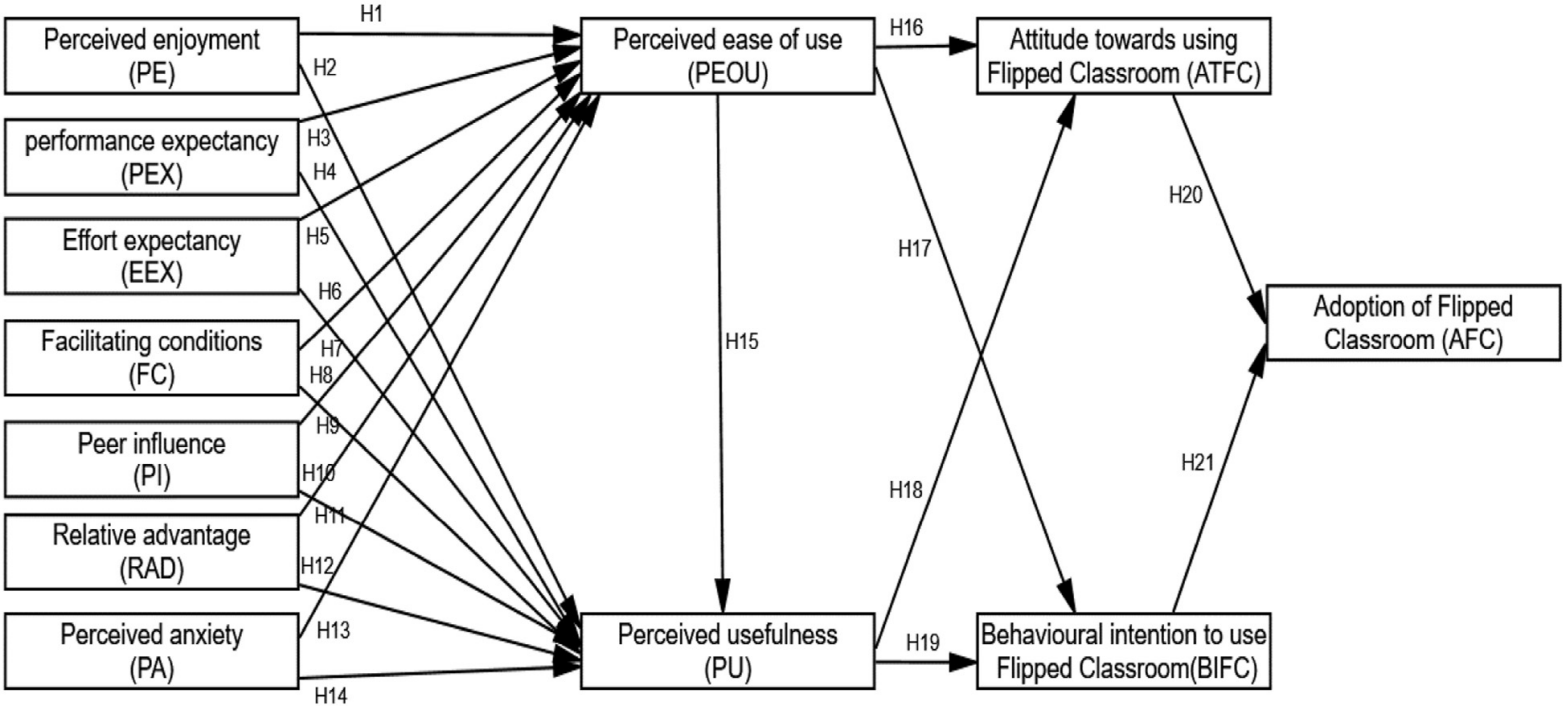
Research model.

Referring technology has had a significant impact on how classroom teaching is delivered and is changing the subject matter. It is impossible to avoid implementing new technologies like virtual reality and innovative teaching strategies like flipping classrooms. The research model in this work is led by the TAM (Davis, 1989) and the Unified Theory of Acceptance and Use of Technology (UTAUT) paradigms. Usefulness, subjective norm, perceived ease of use, and attitude towards using flipped classrooms are investigated by UTAUT, who also takes into account performance expectancy (PEX), effort expectancy (EEX), facilitating condition (FC), and environmental events like perceived anxiety (PA) and relative advantage (ADV). Perceptions of perceived usefulness, perceived ease of use, and social influences (behavioral) on the intention to use Flipped classes are also investigated. While behavioral intent and attitude toward Flipped Learning reflect a person's belief that technology use is manageable and achievable (Davis, 1989), Based on the two aforementioned theories, we selected the linked variables based on perceived anxiety, relative advantage to the aforementioned theoretical framework, study objective, and research situation. Because it is thought that the three variables influence perceived usefulness and perceived usability, respectively, two technological characteristics, reported anxiety and relative advantage, are added. Peer influence (PI), perceived anxiety (PA), perceived ease of use (PEOU), relative advantage (ADV), perceived enjoyment (PE), performance expectation (PEX), effort expectation (EEX), perceived usefulness (PU), perceived ease of use (PEOU), perceived usefulness (PEOU), attitude toward using a Flipped classroom (ATF), and behavioral intention to use a Flipped classroom (BIFC). In the parts that follow, more information is given; refer to Figure 1. To the extant empirical literature, we place emphasis on six essential factors: the theoretical framework (technology anxiety, TA), instructor dimension (ID), course quality (CQ), technology quality (TQ), ease of use (EU), and perceived usefulness (PU). These factors are well-grounded in theories such as the TAM, the ISSM, and the ECT. The study maintains (Sun and Xie, 2020) a classification where TA is classified under the learner dimension (LND), ID is under the instructor dimension (IND), CQ is captured under the course dimension (CSD), TQ under the technology dimension (TGD), and EU and PU under the design dimension (DSD).

### 2.1 Perceived enjoyment (PE)

Perceived enjoyment is the term used to describe the satisfaction or pleasure experienced while using a technology (Davis, 1989). Empirical evidence that demonstrates perceived enjoyment (PE) has a significant influence on the intention to continue using something supports the use of PE as an intrinsic motivation (Venkatesh et al., 2003). Online learning offers learners a variety of fresh and varied learning opportunities, including unique interactive features that make learning more fun and engaging. According to Alrahmi et al. (2021; Luo et al. (2021), users prefer to accept technology that offers enjoyment. According to Van Der Heijden (2004), researchers looked into the connection between consumer intent to return and student contentment (Lin and Lu, 2011). found that the most significant factor influencing SNS usage is enjoyment. There is a connection between students' contentment and their ongoing willingness to use digital books (Lin and Lu, 2011). As a result, if a student finds using internet students' courses online via flipped classroom interesting, they are more likely to want to use web students' online education in the future.

H1 *Perceived enjoyment significantly influences PEOU*.

H2 *Perceived enjoyment significantly influences PU*.

### 2.2 Performance expectancy (PEX)

The performance expectancy factor is described as the "degree to which a person expects that using a system would assist him or her in accomplishing advancements in job performance" (Venkatesh et al., 2003). In this study, "performance expectancy" refers to the extent to which students believe WBI will allow them to finish their out-of-class assignments utilizing the FLCL method. A number of studies (Al-Adwan et al., 2021; Kang et al., 2015; Lakhal et al., 2013; Yeap et al., 2016) demonstrate how performance requirements affect plans to embrace new technologies. This study sought to determine whether there was a relationship between the uptake of TAM technology and the UTAUT component of performance expectations. By examining the effects of effort on the TAM model, notably PU and PEOU, as well as another element, performance expectancy, this study intends to solve the limits of the TAM model, which is an implementation of situationally UTAUT components (Venkatesh et al., 2003).

H3 *Performance expectancy significantly influences PEOU*.

H4 *Performance expectancy significantly influences PU*.

### 2.3 Effort expectancy (EEX)

The effort expectation factor is defined as the "degree of ease connected with the operation of a system" (Venkatesh et al., 2003). In this study, effort expectations are categorized as learners' perceived ease in using a perceived utility, ease of use, attitude towards use, and behavioral control over use. According to several studies (Almaiah et al., 2019; Venkatesh et al., 2003), users' intention to use a specific technology is affected by their expectation of effort. By examining the effects of effort on the TAM model, notably PU and PEOU, as well as another element, effort expectancy, this study intends to address the restrictions of the TAM model, which is a synthesis of context-specific UTAUT components (Venkatesh et al., 2003).

H5 *Effort expectancy significantly influences PEOU*.

H6 *Effort expectancy significantly influences PU*.

### 2.4 Facilitating condition (FCO)

A facilitative condition factor is described as the extent to which a person believes that an organization has evolved to make usage of the system easier (Kissi et al., 2018; Venkatesh et al., 2003; Yang et al., 2019). An enabling condition in this study was defined as the degree to which a student thinks that teachers, families, IT staff, technology, and other decision makers are capable of supporting the use of perceived value, ease of use, attitude toward use, and behavioral control to use in flipped classrooms (Kissi et al., 2018).

H7 *Facilitating condition significantly influences PEOU*.

H8 *Facilitating condition significantly influences PU*.

### 2.5 Peer influence (PI)

Peer influence (PI) is the extent to which students' opinions of their classmates' or coworkers' adoption of technology have affected their choice to do so (Lai and Chen, 2011; Taylor and Todd, 1995). A study on Facebook acceptability among college students found that PI has a positive effect on PU and PEOU (Doleck et al., 2017). According to Hasan et al.e extent to which students' opinions of their classmates' or coworkers' adoption of technology have affected their choice to do so (Lai and Chen, 2011; Taylor and Todd, 1995). A study on Facebook acceptability among college students found that PI has a positive effect on PU and PEOU (Doleck et al., 2017). According to Hasan et al. (2022), however, PI had no impact on the uptake of teaching blogs. According to Zainuddin and Attaran (2016), By using a free classroom management platform to broadcast movies online, flipped classrooms in Thailand are being used to improve the communication skills of EFL learners. This allows students to understand the material and become ready for peer discussion and collaboration exercises in the sections that follow. According to the study, students felt at ease with this kind of instruction, and using this tool during sessions allowed students' communicative skills to increase significantly between pre- and post-tests (Hasan et al., 2022; Lai and Chen, 2011). As a result, PI may contribute to the acceptance and ongoing use of flipped classrooms.

H9 *Peer influence significantly influences PEOU*.

H10 *Peer influence significantly influences PU*.

### 2.6 Relative advantage (RAD)

The degree of creativity that is judged to be better than current concepts is referred to as "relative advantage" (RAD) (Dibra, 2015). It may have to do with a judgment call over whether to use a certain innovation or piece of technology that students deem to be superior to others (Lok, 2015). According to Kosnin et al. (2019), RAD is one of the most important criteria for utilizing LINE, a mobile messaging app, in Saudi Arabia (Doleck et al., 2017). investigated 214 college students' acceptance of flipped courses and discovered that RAD was positively connected to PU. Perceived relative benefits were consistently observed to have a beneficial effect on users' inclination to use the technology across various participants (Lee et al., 2011).

H11 *Relative advantage significantly influences PEOU*.

H12 *Relative advantage significantly influences PU*.

### 2.7 Perceived anxiety (PA)

According to the research paradigm, computer anxiety which is defined as "fear of approaching engagement with a technology that is excessive to the actual harm posed by the computer" is connected to perceived anxiety (Chua et al., 1999; Arpaci and Basol, 2020). Participants with computer anxiety could feel anxious, scared, or hesitant when completing tasks related to the "flipped classroom" (Artino et al., 2010). Anxiety, confusion, and unease can be observed in social interactions, generalizations, and learning experiences as well as in individual encounters with online learning (Trust et al., 2017). It has been discovered that one individual factor influencing system utilization is anxiety. When anxiety levels rise, adaptability and success suffer, according to Venkatesh and Davis (2000) and Zhao et al. (2021). The accessibility of online learning environments has led to a more positive attitude toward students and could have decreased their concern. A personal component that affects how people use systems has been found to be anxiety (Bartimote-Aufflick et al., 2016; Deer et al., 2018). Moreover, Deer et al. (2018) assert that availability and success both decline as anxiety levels grow. The accessibility of online learning environments has led to a more positive attitude toward students and might have decreased their concern.

H13 *Perceived anxiety significantly influences PEOU*.

H14 *Perceived anxiety significantly influences PU*.

### 2.8 Perceived ease of use (PEOU)

Perceived ease of use can be defined as the extent to which an individual thinks using blended learning will be effortless (Davis, 1989). This idea demonstrates the user's assurance that implementing flipped learning would not be challenging. The ease with which skills can be flipped is a good illustration of PEOU. Previous research, for example, has found that the construct has a considerable impact on behavioral attitudes (Phillips and O'Flaherty, 2019). PEOU may also have an impact on the flipping classroom's sustained use. The prior hypothesis states that when technology is perceived as being easy to use, people are more likely to have favorable opinions of it; consequently, the users' evaluations of its utility are evident (Al-Rahmi et al., 2015; Scherer et al., 2019). In addition, individuals are more willing to adopt technology when they see its benefits.

H15 *Perceived ease of use significantly influences PU*.

H16 *Perceived ease of use significantly influences ATFC*.

H17 *Perceived ease of use significantly influences BIFC*.

### 2.9 Perceived usefulness (PU)

According to Davis (1989), the term "degree to which the individual believes that adopting a technology system will increase his or her job achievement" refers to the degree to which a person feels that using a specific system would boost work performance. The perceived utility of flipping classrooms can be defined as the degree to which a person believes that it can be a driving element toward achieving learning objectives (Mohamed and Lamia, 2018). Therefore, this is regarded as the extent to which a user comprehends that employing a flipped classroom might improve a user's learning system. PU, according to Al-Rahmi et al. (2015) and Huang et al. (2019), is a concept that has been shown to have an impact on attitude. Similarly, PU may influence users' intentions to use the flipped classroom in the future; therefore, users' views of its utility are visible. Additionally, students are more passionate about embracing technology when they perceive it to be useful (Al-Rahmi et al., 2015; Scherer et al., 2019).

H18 *Perceived usefulness significantly influences ATFC*.

H19 *Perceived usefulness significantly influences BIFC*.

### 2.10 Attitude towards using flipped classroom (ATFC)

The degree to which a person experiences a positive or negative sensation associated with flipping classrooms has been defined as their attitude toward using them (Chang et al., 2015; Hsi-Peng and Hsiao, 2007). Many earlier empirical investigations (Ifinedo, 2018) revealed that attitudes toward flipped classrooms, positive learning benefits, and intention to utilize them were all major factors in the decision to use those (Vanduhe et al., 2020). As a result, how much a user conveys a positive or negative sensation in the system can be interpreted as their attitude toward using Flipped Classroom? In a related vein, Al-Rahmi et al. (2015) and Scherer et al. (2019) argued that the TAM's relationship between attitude and intention suggests that attitude functions as a behavioral observation attitude.

H20 *Attitude towards using Flipped classroom significantly influences ATFC*.

### 2.11 Behavioral intention to use flipped classroom (BIFC)

According to Venkatesh et al. (2003), behavioral intention to use is identified as the learners' decision whether to continue using the system or not, and this term is seen as a factor influencing the usage of a technology. Therefore, the BPBL strategy is anticipated to be able to improve students' educational success through behavioral intention (Khlaisang et al., 2021) in this study. All of these theories and models are based on the core ideas of TRA, behaviors, and subjective standards that consider web-based learning as a function. Online learning and control beliefs were added to UTAUT and TAM (Davis, 1989; Venkatesh et al., 2003). The most likely predictors of reported ease of use and perceived usefulness were found to be higher levels of continued intention and user satisfaction (Davis, 1989; Venkatesh et al., 2003). The goal of this study was to determine the extent to which students believe that using the online learning strategy increases their acceptance of flipped classrooms.

H21 *Behavioral intention to use flipped classrooms significantly influences BIFC*.

### 2.12 Adoption of flipped classrooms (AFC)

The Flipped Classroom refers to how much a person believes his or her institution's foundation supports his or her adoption and utilization of Flipped Classroom technologies (Unal and Unal, 2017). An intriguing justification for the popularity of the flipped classroom method in education is its adaptability to support students' active learning, collaboration, and mentoring during the learning opportunity by effectively arranging teaching time (Kim et al., 2019; Sergis et al., 2018). As a result, when advance organizers are used, face-to-face sessions become more student-centered, with time set aside for discussion, group discussions, and problem-solving (Pickering and Roberts, 2018). The possibility of changing the teacher's position to one of a facilitator and guide is another exciting feature that highlights the value of adopting flipped classrooms (Lopes and Soares, 2018). Additionally, pre-class assignments, which students are expected to finish before the start of actual class time, can be a way for material to be transferred outside of the classroom (Miller et al., 2018). That is, rather than lecturing, class time might be spent on giving learners unique learning experiences through cooperative groups with their classmates and getting assistance from their teacher (DeLozier and Rhodes, 2017). The acceptance of faculty members in Saudi Arabian universities toward the adoption of flipped classroom-based technology was investigated in this study. The flipped classroom-based technology leads to a student-centered approach that focuses on active learning, problem-solving skills, higher-order thinking, and the participation of the students in group discussions.

## 3 Research methodology

### 3.1 The study's design

The study's main purpose was to develop a simple and easy-to-understand theoretical model for investigating flipping the classroom's acceptability and its factors. A multistage testing strategy was used to construct, validate, and test the suggested model and survey. To begin, participants tested sixty (60) previously used questions to measure Flipped classroom acceptance during COVID-19 at Saudi institutions, extracting ten components to evaluate Flipped classroom acceptance during COVID-19. Second, approval for ethical clearance was obtained for this study (Ref. No. KFU-REC-2022-AUG-ETHICS116), and data was collected from 208 students, both online and manually, who were chosen at random from King Faisal University students in Saudi Arabia. Collected data were evaluated with IBM SPSS (version-26), and the partial least square structural equation model (PLS-SEM3.3.3). The questionnaire's items, which included elements of the assessment TAM model and UTAUT theory with external factors, were scored using a 5-point Likert scale. Structural equation modeling (SEM) (Davis, 1989) was utilized in the empirical study to empirically evaluate the proposed conceptual model of the adoption of flipped classrooms during COVID-19. In order to validate the results and show the significance of the outcomes in this investigation, appropriate statistical tests were used. The survey was carried out in person, and participants were invited to return it once it was finished. The survey focused on respondents' attitudes towards using Flipped Classrooms, as well as their behavioral intentions to use them and their perceptions of their impact on Flipped Classroom adoption during COVID-19. SPSS was used for data analysis together with structural equation partial least squares modeling for the data inquiry (Smart PLS 3.3.3).

### 3.2 Data collection

The King Faisal University undergraduate students in Saudi Arabia are the study's target group. The data collection procedure included the use of a questionnaire survey. The poll included a total of 225 participants. As a result, the sample size used in this study is adequate to capture Saudi Arabian students' perceptions of the implementation of flipped classrooms. Twelve polls in all were excluded because of missing data. A total of 213 questionnaires were distributed, and 225 of them, representing a return rate of 94.6%, were returned by respondents. A questionnaire survey with a quantitative methodology was used in this investigation. Students at King Faisal University were issued self-administrated questionnaires between May and July 2022 in order to gather the data. Thus, from a total of 213 questionnaires, the data was analyzed using SPSS. The researcher discussed the study's objectives and the description of online meetings, such as Google Meet, at the start of the data collection process, and the students then responded to the questionnaire. Table 1 shows the gender, age, specialization, and frequency of use of flipped classrooms among the respondents. According to the survey's demographics, females made up 57 (26.8%) of the respondents, while males made up 156 (73.2%). 40 (18.8%) of those polled were between the ages of 18 and 20. 104 (40.8%) of those who responded were between the ages of 21 and 24. 54 (25.4%) of those polled were between the ages of 25 and 29. Twelve (5.6%) of those polled were between the ages of 30 and 34. Finally, 3 (1.4% of respondents) were 35 years old or older.

**Table 1:** Demographic profile.

Demographic	Discerption	N	%
Gender	Male	156	73.2
	Female	57	26.8
Age	18–20	40	18.8
	21–24	104	48.8
	25–29	54	25.4
	30–34	12	5.6
	45 and Above	3	1.4
Specialization	Management	96	45.1
	Science & Technology	57	26.8
	Engineering	34	16.0
	others	26	12.2
Use flipped classrooms	I am currently using it	210	98.6
	I haven't use it	3	1.4

### 3.3 Instrument creation

Data was collected using a five-point Likert scale with the following options: (1) Strongly Disagree, (2) Disagree, (3) Natural, (4) Agree, and (5) Strongly Agree. The present investigational hypotheses were applied with the following modifications to fit the study's framework: Perceived enjoyment (PE) was adapted from the sample questionnaire from (Abdekhoda et al., 2020; Luo et al., 2021), performance expectancy was adapted from the sample questionnaire from (Almaiah et al., 2019; Kissi et al., 2018), effort expectancy was adapted from (Almaiah et al., 2019; Kissi et al., 2018), facilitating conditions was adapted from the sample survey from (Khlaisang et al., 2021), and Perceived anxiety was adapted from the sample survey from Al-Emran and Teo (2020; Davis et al., 1989), and attitude towards us was adapted from the sample questionnaire from Abdekhoda et al. (2020; Khlaisang et al., 2021). Finally, during COVID-19, the adoption of flipped classrooms was modified from a sample questionnaire (Kim et al., 2019). Table 3 lists all characteristics and their resources.

## 4 Research analysis

### 4.1 Models of measurement

The "model fit" refers to the evaluation procedures that check the accuracy and validity of the measurements. There were three separate statistics used. Discriminant validity, convergent validity, indicator load conditions, and internal consistency reliability are the three categories of validity (Hair et al., 2019). also recommends three metrics to use.

#### 4.1.1 Reliability coefficient, indicator loadings, and internal reliability

Results from PLS-SEM were used in this investigation to determine indicator loadings. The details of the loadings are displayed in Table 3. Most of the items were in compliance with the suggested loading values of > .700 (Hair et al., 2019). In the second stage of the PLS-SEM study, fifty indicators were assessed. Internal consistency reliability refers to the evaluation results for statistical uniformity across indicators. Internal consistency reliability should be measured using Cronbach's alpha and converging validity. According to the criteria outlined by Hair et al. (2019), validity scales should be > .700; the Cronbach's alpha and converging validity coefficients in this study were calculated using this criterion. Table 2 highlights the details of the two measured data sets. Strong internal consistency is shown for all variables by Cronbach's alpha and converging validity values, which range from 0.856 to 0.929 for Cronbach's alpha reliability and from 0.897 to 0.946 for composite reliability.

**Table 2:** Reflective indicator loadings, internal consistency reliability, and convergent validity.

Construct	Load	Alpha	Composite Reliability	AVE
Perceived enjoyment (PE)	0.844	0.908	0.932	0.732
	0.892			
	0.840			
	0.877			
	0.822			
Performance Expectancy (PEX)	0.800	0.890	0.919	0.696
	0.849			
	0.887			
	0.870			
	0.759			
Effort Expectancy (EEX)	0.721	0.864	0.902	0.650
	0.731			
	0.826			
	0.850			
	0.890			
Facilitating conditions (FC)	0.745	0.880	0.913	0.678
	0.862			
	0.854			
	0.849			
	0.801			
Peer influence (PI)	0.830	0.897	0.924	0.709
	0.845			
	0.870			
	0.868			
	0.795			
Relative advantage (RAD)	0.703	0.916	0.939	0.756
	0.944			
	0.859			
	0.869			
	0.951			
Perceived anxiety (PA)	0.900	0.929	0.946	0.779
	0.870			
	0.903			
	0.896			
	0.843			
Perceived ease of use (PEOU)	0.908	0.895	0.923	0.708
	0.832			
	0.858			
	0.876			
	0.722			
Perceived usefulness (PU)	0.758	0.856	0.897	0.637
	0.721			
	0.799			
	0.852			
	0.852			
Attitude towards using Flipped classrooms (ATF)	0.875	0.924	0.943	0.767
	0.901			
	0.894			
	0.875			
	0.832			
Behavioural intention to use Flipped Classroom(BIFC)	0.848	0.910	0.933	0.735
	0.869			
	0.884			
	0.845			
	0.839			
Adoption of Flipped Classroom (AFC)	0.731	0.867	0.904	0.654
	0.833			
	0.863			
	0.792			
	0.819

**Table 3:** Fornell-Larcker criterion.

	PEOU	AFC	ATF	BIFC	EEX	FC	PI	PA	PE	PU	PEX	RDV
PEOU	0.841											
AFC	0.683	0.809										
ATF	0.626	0.603	0.876									
BIFC	0.671	0.617	0.526	0.857								
EEX	0.765	0.743	0.786	0.653	0.806							
FC	0.712	0.728	0.648	0.679	0.746	0.824						
PI	0.603	0.586	0.478	0.489	0.582	0.499	0.842					
PA	0.676	0.680	0.550	0.554	0.685	0.636	0.745	0.883				
PE	0.722	0.765	0.564	0.584	0.716	0.634	0.594	0.681	0.855			
PU	0.776	0.803	0.634	0.671	0.782	0.761	0.713	0.802	0.827	0.798		
PEX	0.650	0.719	0.653	0.627	0.812	0.675	0.742	0.836	0.663	0.752	0.834	
RDV	0.736	0.729	0.605	0.645	0.714	0.732	0.622	0.701	0.699	0.903	0.702	0.870

#### 4.1.2 Validity convergence

Convergent validity is a quantitative problem related to construct validity. Evaluations utilizing the same or similar conceptions should be substantially connected, according to the theory of model fit. The AVE scores must be given in terms of composite reliability. The smart PLS scores were calculated using a PLS-SEM technique. The AVE score should be greater than or equal to 500, and it should explain at least 50% of the variance. The AVE value for all buildings is greater than or equal to 500, which accounts for more than half of the difference (Table 2).

#### 4.1.3 Validity that discriminates

According to Hair et al. (2019), exceeds 900discriminant validity is a construct's ability to distinguish itself from other constructs. The Fornell-Larcker criterion requires that the AVE value of a construct be less than the shared variance for all of the model's constructs. The results of the investigation show that the AVE scores of each component are less than their common variance (Fornell and Larcker, 1981) (Table 2). Therefore, a revcriterione Criterion demonstrated discriminant validity. Cross-loadings can also be utilized taluate the discriminant valitheity. When a loaofng value on a concept is greater than all of its pass values on other ideas, discriminant validity occurs. All of the indicators' factor loading values (in bold) for each structure, as shown in Table 3, were higher than values pass value for the other constructions. Discriminant validity developed as a result of the examination of cross-loading value. When the HTMT reading exceeds.900 If HTMT has a value greater than 0.900, the concept does not have discriminant validity. All of the HTMT readings in Table 4 were less than 900. The results show that the values were substantially different from 1.

**Table 4:** Heterotrait–monotrait (HTMT).

	PEOU	AFC	ATF	BIFC	EEX	FC	PI	PA	PE	PU	PEX
PEOU											
AFC	0.772										
ATF	0.689	0.671									
BIFC	0.742	0.692	0.574								
EEX	0.840	0.848	0.888	0.725							
FC	0.798	0.828	0.715	0.758	0.842						
PI	0.671	0.662	0.523	0.536	0.645	0.555					
PA	0.740	0.756	0.592	0.600	0.753	0.695	0.816				
PE	0.797	0.858	0.614	0.639	0.790	0.702	0.658	0.742			
PU	0.886	0.828	0.711	0.756	0.892	0.870	0.822	0.804	0.834		
PEX	0.727	0.817	0.719	0.696	0.823	0.758	0.826	0.920	0.738	0.862	
RAD	0.817	0.818	0.659	0.709	0.795	0.813	0.684	0.757	0.763	0.710	0.778

### 4.2 Goodness of fit criterion

In recent times, a proper general measurement has been suggested for the overall model fit with PLS utilization. The amount obtained for it is between 0 and 1 (Wetzels et al., 2009). have presented three amounts of 0.01, 0.25, 0.36 as a weak, medium, and strong GOF amounts. In other words, if we compute 0.01 and its closest amount as GOF in a model, we can say that the overall model fit is weak, and we require to correct the connections among model constructs. On the other side, this guideline is confirmed, and the GOF computation formula is as below in the presence of the other two GOF amounts (0.25: moderate overall fit, 0.36: strong overall fit) (Wetzels et al., 2009). The model fit was good, with items demonstrating strong model fit (χ2 = 6264.870; CFI = 0.915; SRMR = 0.070).

### 4.3 Evaluation of a structural model

There are certain steps in the structure model evaluation (Hair et al., 2019). With the presentation of Variance Inflation Factor (VIF) numbers, correlation became more computerized. In the second stage, the link was investigated. In step three, the level of significance (R2) was computed. The effect size of f2 for the construct's relevancy was reported in step four, which had the objective of examining the rationale behind the chosen endogenous latent variables. The data were examined in PLS-SEM using the blinding technique for the R2 and f2 effect sizes in addition to providing the Q2 values.

#### 4.3.1 Validity that discriminates

Collinearity between the sets of variables should be investigated. The collinearity is determined by looking at the VIF value. If the VIF value is stated to be > 3.000, cointegration will be a concern (Hair et al., 2019). Attitude toward using flip classrooms (ATF) influences acceptance of flip classrooms (AFC) (VIF = 1.383); effort expectancy (EEX) influences perceived ease of use (PEOU) (VIF = 1.299) and perceived usefulness (PU) (VIF = 1.959); and behavioral intention to use flip classrooms (BIFC) influences adoption of flip classrooms (VIF = 1.959). The confidence intervals are all less than three (Table 5). Therefore, collinearity is not a problem in our investigation because all factor loadings are less than 3 (Hair et al., 2019).

**Table 5:** Variance inflation factor (VIF).

	PEOU	AFC	ATF	BIFC	EEX	FC	PI	PA	PE	PU
PEOU			2.516	2.516						2.557
AFC										
ATF		1.383								
BIFC		1.383								
EEX	1.299									1.959
FC	2.827									2.934
PI	2.658									2.745
PA	2.224									2.340
PE	2.634									2.745
PU			2.516	2.516						
PEX	1.768									2.162
RAD	2.101									2.225

#### 4.3.2 Testing hypotheses

**Figure 2:**
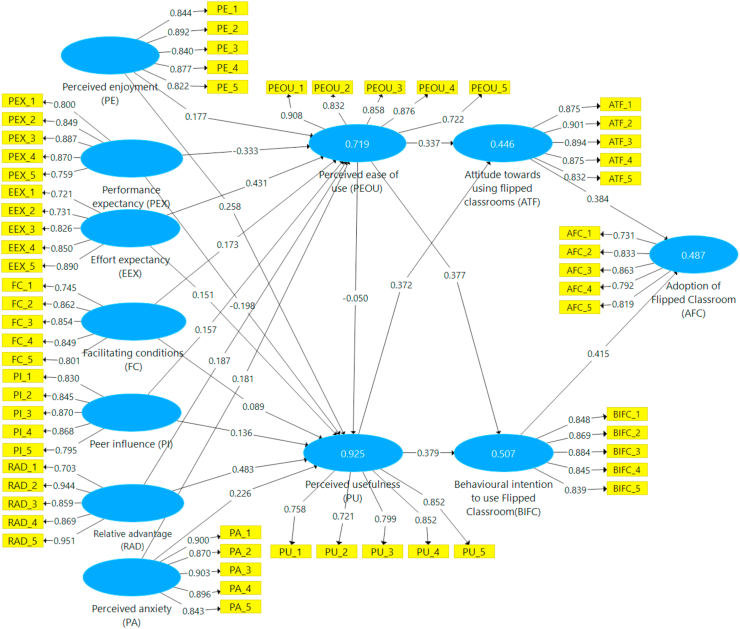
The findings for path coefficient.

**Figure 3:**
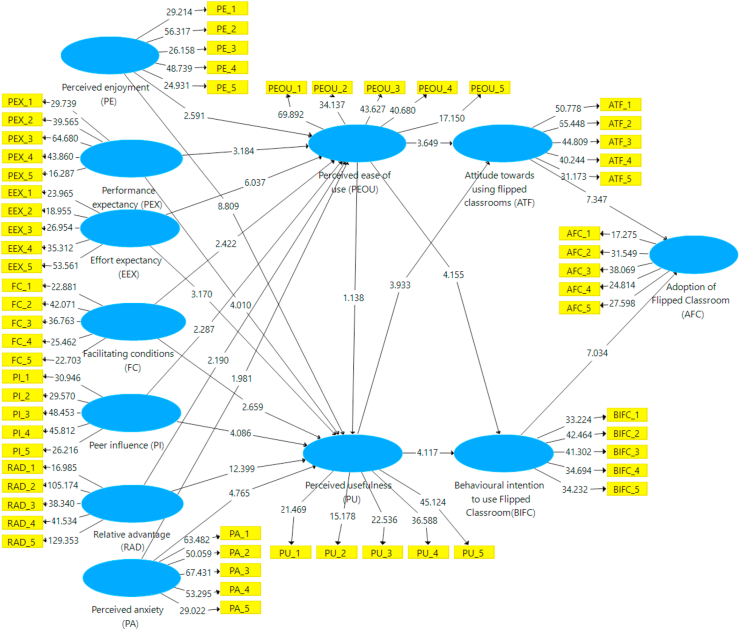
Path (T-values) findings.

The company constructed the sample using 5,000 subsamples in order to calculate the route coefficient between endogenous and exogenous structures. Figure 1 depicts the hypothesis, Figure 2 the results of the path coefficient, and Figure 3 the outcomes of the path (T-values). In this work, SEM analysis was used to evaluate the research model. Twenty-one hypotheses were tested in the research model to investigate the effect of the TAM model and UTAUT theory on attitudes towards using flip classrooms and behavioral intentions to use flip classrooms (BIFC), which can affect flip classroom implementation for teaching and learning. The results validated twenty hypotheses, while one hypothesis was rejected in the research model, as shown in Table 6. Perceived enjoyment has a beneficial influence on students' perceptions of usefulness (β = 0.258, t = 8.809) and perceived ease of use (β = 0.177, t = 2.591). Therefore, H1 and H2 have statistical significance. Performance expectancy (PEX) had a significant positive influence on perceived usefulness (β = −0.198, t = 4.010) and perceived ease of use (β = 0.333, t = 3.184) for the flipped classroom approach. Thus, H3 and H4 were statistically significant. According to Table 6, the "flipped classroom" strategy had a significant positive influence on perceived usefulness (β = 0.151, t = 3.170) and perceived ease of use (β = 0.431, t = 6.037). Therefore, H5 and H6 have statistical significance. Furthermore, with the flipped classroom approach, the facilitating condition (FC) had a significant positive influence on perceived usefulness (= 0.089, t = 2.659) and perceived ease of use (β = 0.173, t = 2.422). Therefore, H7 and H8 have statistical significance. Table 6 shows a significant positive relationship between peer influence on perceived usefulness (β = 0.136, t = 4.086) and perceived ease of use (β = 0.157, t = 2.287).

**Table 6:** Structural model for hypothesis testing results.

H	Hypotheses	Β	T Values	P Values	Results
H1	Perceived enjoyment (PE) ------> Perceived usefulness (PU)	0.258	8.809	0.000	Supported
H2	Perceived enjoyment (PE) ------> Perceived ease of use (PEOU)	0.177	2.591	0.010	Supported
H3	Performance Expectancy (PEX) ----> Perceived usefulness (PU)	−0.198	4.010	0.000	Supported
H4	Performance Expectancy (PEX) ----> Perceived ease of use (PEOU)	−0.333	3.184	0.002	Supported
H5	Effort Expectancy (EEX) ----> Perceived usefulness (PU)	0.151	3.170	0.002	Supported
H6	Effort Expectancy (EEX) ----> Perceived ease of use (PEOU)	0.431	6.037	0.000	Supported
H7	Facilitating conditions (FC) ----> Perceived usefulness (PU)	0.089	2.659	0.008	Supported
H8	Facilitating conditions (FC) ----> Perceived ease of use (PEOU)	0.173	2.422	0.016	Supported
H9	Peer influence (PI) ----> Perceived usefulness (PU)	0.136	4.086	0.000	Supported
H10	Peer influence (PI) ----> Perceived ease of use (PEOU)	0.157	2.287	0.023	Supported
H11	Relative advantage (RAD) ----> Perceived usefulness (PU)	0.483	12.399	0.000	Supported
H12	Relative advantage (RAD) ----> Perceived ease of use (PEOU)	0.187	2.190	0.029	Supported
H13	Perceived anxiety (PA) ----> Perceived usefulness (PU)	0.226	4.765	0.000	Supported
H14	Perceived anxiety (PA)----> Perceived ease of use (PEOU)	0.181	1.981	0.048	Supported
H15	Perceived ease of use (PEOU) ----> Perceived usefulness (PU)	−0.050	1.138	0.256	unsupported
H16	Perceived ease of use (PEOU) ----> Attitude towards using Flipped classrooms (ATF)	0.337	3.649	0.000	Supported
H17	Perceived ease of use (PEOU) ----> Behavioural intention to use Flipped Classroom(BIFC)	0.377	4.155	0.000	Supported
H18	Perceived usefulness (PU) ----> Attitude towards using Flipped classrooms (ATF)	0.372	3.933	0.000	Supported
H19	Perceived usefulness (PU) ----> Behavioural intention to use Flipped Classroom(BIFC)	0.379	4.117	0.000	Supported
H20	Attitude towards using Flipped classrooms (ATF) ----> Adoption of Flipped Classroom (AFC)	0.384	7.347	0.000	Supported
H21	Behavioural intention to use Flipped Classroom(BIFC) -----> Adoption of Flipped Classroom (AFC)	0.415	7.034	0.000	Supported

As a result, the H9 and H10 hypotheses are validated. Table 6 shows that adopting flipped classrooms for learning has a significant positive association with perceived usefulness (β = 0.483, t = 12.399) and perceived ease of use (β = 0.187, t = 2.190). The findings also show a positive relationship between relative advantage (RAD) and perceived usefulness (PUE) with perceived ease of use learning. Thus, H11 and H12 were supported. Similarly, the thirteenth and fourteenth hypotheses hypothesized that perceived anxiety (PA) influenced perceived usefulness (H13 = 0.226, t = 4.765) and perceived ease of use for flipped classrooms (H14 = 0.181, t = 1.981). The hypotheses (H13 and H14) are therefore confirmed. Furthermore, Table 6 shows a negative relationship between the perceived usefulness and ease of use of flipped classrooms for learning (H15 = −0.050, t = 1.138). This means that TAM was unaffected by the gender of the students. The assumptions of a link between perceived ease of use and attitude toward utilizing flip classrooms (ATF) (H16 = 0.337, t = 3.649) and actual flip classroom behavior (H17 = 0.377, t = 4.155) were also tested. As a result, the hypotheses (H16 and H17) were supported. As shown in Table 6, the hypotheses H18 and H19, which assumed a positive and significant relationship between perceived usefulness and attitude towards using flipped classrooms (H18 = 0.372, t = 3.933) and behavioral intention to use flipped classrooms (H19 = 0.379, t = 4.117) for utilizing flipped classrooms, Thus, the hypotheses were validated. According to Table 6, attitude toward using flipped classrooms was positively connected to adoption of flipped classrooms (H5 = 0.384, t = 7.347). As a result, H20 was accepted. Finally, the hypothesis of a link between behavioral intention to utilize Flipped Classroom and Flipped Classroom adoption (H21 = 0.415, t = 7.034) was tested. As a result, H21 has statistical significance. Table 6 shows the results.

#### 4.3.3 Determination coefficient (R2)

The determination coefficient (R2), the result of regression analysis, is defined as the variable percent in the predictor variables that may be anticipated by the exogenous variable. It assesses a suggested model's predictive accuracy. It's calculated as the square of the correlation between two endogenous constructs. The R2 scale runs from 0 to 1, with a greater value indicating a higher level of R2. A value of 0.25 is considered weak, 0.50 is considerable, and 0.75 is significant (Hair et al., 2019) Table 7 presents the R2 result based on the study's findings. PEOU (Perceived Ease of Use) (0.719, Average), AFC (Adoption of Flipped Classroom) (0.487, Average), ATF (Attitude Towards Flipped Classrooms) (0.446, Average), BIFC (Behavioral Intention to Use Flipped Classroom) (0.507, Average), and PU (Perceived Utility) (0.925, High). Table 7 shows the results.

**Table 7:** Coefficient of determination (R2).

	R Square	Results
Perceived ease of use (PEOU)	0.719	moderate
Adoption of Flipped Classroom (AFC)	0.487	moderate
Attitude towards using Flipped classrooms (ATF)	0.446	moderate
Behavioural intention to use Flipped Classroom(BIFC)	0.507	moderate
Perceived usefulness (PU)	0.925	substantial

#### 4.3.4 Determination coefficient (R2)

The strength of a predictive construct's relationship with an explained variable is quantified by the statistical notion known as the effect size, or f2. In other words, f2 is utilized to evaluate how exogenous constructs affect endogenous latent variables. Whenever an exogenous variable is taken out of the model, F2 examines how well the R2 value changes. According to Hair et al. (2019), an f2 value of 0.02 has a minor impact, a value of 0.15 has a moderate impact, and a value of 0.35 has a significant impact. Seven confirmatory factor effect sizes were discovered in the study's data. The relationship between perceived ease of use (PEOU) and attitude toward using flipped classrooms (ATF) had the largest effect (0.481), whereas the relationship between PEOU and behavioral intention to use flipped classrooms (BIFC) had a medium f2 value of 0.314 (See Table 8).

**Table 8:** f2 result.

	F^2^	Effect size		F^2^	Effect size
PEOU---------- > ATF	0.481	Large	RAD--------> PEOU	0.440	Large
PEOU---------- > BIFC	0.314	Medium	RAD--------> PU	0.969	Large
PEOU---------- > PU	0.010	No effect	PI---------- > PEOU	0.333	Medium
ATF---------- > AFC	0.208	Medium	PI---------- > PU	0.291	Medium
BIFC---------- > AFC	0.242	Medium	PA--------- > PEOU	0.127	Small
EEX---------- > PEOU	0.253	Medium	PA---------- > PU	0.147	Small
EEX---------- > PU	0.562	Large	PE--------- > PEOU	0.242	Medium
FC ---------- > PEOU	0.338	Medium	PE--------- > PU	0.125	mall
FC ---------- > PU	0.436	Large	PU--------- > ATF	0.399	Large
PEX---------- > PEOU	0.268	Medium	PU--------- > BIFC	0.216	Medium
PEX---------- > PU	0.585	Large			

## 5 Research discussion, and implications

As shown in Figure 1, this study used the TAM and UTUAT with external variables to evaluate factors that influence students' attitudes toward utilizing flipped classrooms and their desire to utilize flipped classrooms in higher education in Saudi Arabia. Students at King Faisal University provided the data used in this study's research. The results of the structural equation modeling analysis in the study back up 20 of the 21 research hypotheses listed in Table 6. Perceived enjoyment, effort expectancy, performance expectancy, a facilitating situation, peer influence, facilitating conditions, perceived anxiousness, perceived usefulness, usefulness and ease of use, mindset toward using blended learning, actual conduct to use blended learning, and implementation of classroom instruction in Saudi university education are all included in the TAM with UTAUT and situational factors in the study model. As a result, the research methods identify TAM with UTAUT as the most significant influences on the adoption of flipping classrooms in terms of behavioral intention to use flipping classroom environments as an educational strategy and attitude toward using flipping classrooms. As a result, the study's findings strongly support the perceived enjoyment variable, supporting hypotheses (H1 and H2) that perceived enjoyment influences perceived usefulness and usability favorably. This is consistent with earlier studies, which showed a positive correlation between reported satisfaction and perceived usefulness and usability (Al-rahmi et al., 2021, Al-Rahmi et al., 2021; Alasmari and Zhang, 2019; Dibra, 2015; Gündüz and Akkoyunlu, 2019; Luo et al., 2021; Shahnama et al., 2021). Thus, the results of the study provide strong evidence in favor of the performance expectation variable, validating the hypotheses (H3 and H4) and showing that performance expectancy positively affects perceived usefulness and perceived usability. This is in line with earlier studies that discovered a link between perceived utility and simplicity of use and reported satisfaction with usability (Dibra, 2015; Gündüz and Akkoyunlu, 2019; Luo et al., 2021). The study's results also show that effort expectation has a positive effect on perceived usefulness and perceived ease of use, validating hypotheses (H5 and H6) and providing strong evidence for this claim. To put it another way, when a flipped classroom is easy to use and accept, the higher effort expectation leads to more use of the perceived utility and, therefore, perceived ease of use. Thus, this is in line with earlier studies that found a strong correlation among effort expectancy and perceived usefulness and usability (Almaiah et al., 2019; Luo et al., 2021).

The second variable is facilitating conditions, which has two hypotheses (H7 and H8) that significantly benefited students' perceptions of the utility and simplicity of adopting a flipped classroom. According to earlier studies, being directly or indirectly engaged had a favorable correlation with perceived value and usability (Agyei and Razi, 2022; Almaiah et al., 2019). This is consistent with those findings. The study's findings largely support the peer impact characteristic, supporting hypotheses (H9 and H10) that peer pressure influences perceptions of usefulness and usability in a positive way. To put it another way, more peer influence results from perceived usefulness and convenience of use being more widely utilized when a classroom is straightforward to use and accepted. This is in line with earlier studies that found peer influence has a positive relationship with perceived utility and usability (Salajan et al., 2015; Zhou et al., 2019). These results, however, were at odds with those of earlier research (Almaiah et al., 2019; Khlaisang et al., 2021). Relative advantage, the second variable, had alternative hypotheses (H11 and H12) that significantly improved how students perceived the usefulness and ease of using flipped classes in higher education. This is consistent with earlier studies, which showed a favorable correlation between perceived utility, perceived usability, and relative advantage (Lee et al., 2011; Luo et al., 2021). The study's findings also wholeheartedly support the felt anxiety variable, validating hypotheses (H13 and H14) that experienced anxiety influences perceived usefulness and usability favorably. This is consistent with other studies, which showed that usefulness and stated ease of use were positively correlated with felt anxiety (Arpaci and Basol, 2020; Zhao et al., 2021).

The results of the study refuted the ease of use factor, supporting hypothesis (H15) that perceived ease of use had no positive effect on perceived utility (Al-Emran and Teo, 2020). The next parameter is considered ideal for use. These results, however, were at odds with those of earlier research (Al-Rahmi et al., 2022b; Cai et al., 2019; Luo et al., 2021). Research theories (H16 and H17) related to the next factor, perceived usefulness and ease of use, had a significant beneficial impact on attitudes about using flipped classrooms as well as intentions to utilize flip courses in higher education. This is in line with an earlier study, which discovered a favorable relationship between relative advantages and beliefs about using flipped classes as well as actual behavior in flipped classrooms (Al-Rahmi et al., 2022a; Gómez-García et al., 2020). These results, however, ran counter to those of an earlier study (Mohamed and Lamia, 2018). The results of the study wholeheartedly support the usefulness variable, verifying theories (H18 and H19), showing that perceived usefulness has a favorable impact on attitudes toward using blended learning and behavioral intentions to use flip classrooms. The next parameter is perceived usefulness. This is consistent with previous research that found a link between perceived usefulness, attitudes toward using flipped classes, and actual behavior to use flipped classrooms (Abdekhoda et al., 2020; Vanduhe et al., 2020). The next factor is students' attitudes toward adopting flipped classrooms, which had one hypothesis (H20) with significant positive effects on their uptake in higher education. This is consistent with an earlier study, which showed a positive correlation between opinions toward using flipped classrooms and their use in university education (Khlaisang et al., 2021; Ullah et al., 2021; Vanduhe et al., 2020). The behavior intention to use reversed classrooms parameter, which shows that theory of planned behavior to use blended learning positively affects adoption of reversed classrooms, finally verifies hypothesis (H21), which is consistent with earlier studies that showed a positive correlation between students' behavioral intentions to utilize flipped learning and the uptake of flipped learning in higher education (Abdekhoda et al., 2020; Khlaisang et al., 2021; Kim et al., 2019).

### 5.1 Theoretical and practical implications

Based on the model and findings, this research adds to the body of knowledge by suggesting a model that unifies the unified theory of acceptance and usage of technology (UTUAT) theory with the technology acceptance model (TAM) model, demonstrating a useful model to recognize those four key implications. First, the effects of the flipped classroom on perceived satisfaction, performance expectations, effort expectations, enabling conditions, peer influence, relative advantage, and perceived anxiety increased student adoption of flipped classrooms due to perceived value and usability (AFC). Second, perceived utility and perceived usability of flipped classrooms affect attitudes toward flipped learning and behavioral intentions to use flipped learning, which enhance the adoption of flipped learning (AFC). Third, a person's attitude toward using flipped classrooms and their behavioral desire to do so It also aims to boost higher education's adoption of flipped classrooms. Next, a theoretical model for flipped classrooms using TAM, UTAUT, and other related technologies is being developed. The study's contribution to the first model combines the unified theory of acceptance and utilization of technology (UTUAT) theory with the technology acceptance model (TAM) model. Future flipped classrooms can also be used to improve learning and teaching results by using the technology acceptance approach.

By answering the research questions, the study's key practical ramifications and contributions are thus attained. First, the technology acceptance model confirmed that it is a suitable model for achieving independent variables to enhance students' perceptions of the usefulness, usability, attitude, and behavioral intention of using flipped classrooms, which enhances their adoption of flipped classrooms in higher education. Second, the unified theory of acceptance and usage of technology provides evidence to model independent variables and to increase perceived usefulness, perceived ease of use, attitude toward using flipped classrooms, and behavioral intention to use flipped classrooms. As a result, there has been an increase in student adoption of flipped classrooms in higher education. Thus, this study makes important theoretical contributions to earlier research in these areas, which did not previously identify the effects of using flipped classrooms on perceived utility, perceived simplicity, attitude toward using flipped classrooms, and behavioral intention to use flipped classrooms (Agyei and Razi, 2022; Bakheet and Gravell, 2020).

### 5.2 Model limitations

Although this analysis shows that statistical support is available, it has a number of flaws. There are some issues with this research. The first is that the research topic is restricted to what one Saudi Arabian university is capable of covering. Future research would need more participants from a variety of majors because every response in this collection came from the same university. The sample relied on students' expectations, which can differ from teachers' opinions because it lacked qualitative information. Future studies are advised to replicate the research in other provinces besides Saudi Arabia, where the environment is different, and take these limitations into further consideration. It is also recommended that additional follow-up experimental and mixed-methods research be conducted in this area of study.

## 6 Conclusion

Through the use of TAM and UTAUT as guiding theories, as well as relative advantage and perception anxiety as extrinsic factors, this study aimed to examine the factors that influence perceived usefulness and ease of use, attitude towards the use of blended learning, and actual behavior for flipped learning adoption. Perceived enjoyment, effort expectancy, spatial arrangements, peer influence, facilitating conditions, and perceived anxiety are all predictors of perceived usability, perceived ease of use, a mindset toward using blended learning, and behavioral control to use blended learning among Saudi university students. The results also demonstrate that behavioral intentions to adopt blended learning and attitudes about using blended learning have an effect on the adoption of classroom instruction for educational learning. Additionally, perceived ease of use has a negative impact on perceived utility. They also discovered that perceived usefulness, perceived usefulness and ease of use, attitudes toward the use of flipped classrooms, and behavioral intentions to use flipped classrooms are all important for long-term sustainable development in higher education. Attempting to combine TAM and UTAUT with external factors such as competition chance and perceived anxiety had a significant impact on the study's outcome. This investigation has a variety of implications in light of the model and findings. The first result is the worth of predetermined structures. In the function of usability and usefulness, the positive relationship between perceived pleasure, achievement anticipation, effort expectancy, the facilitative condition, peer influence, facilitating conditions, and perceived anxiety is crucial. Second, instructors can illustrate how to use flipped classrooms by giving students instructional tools to aid in their understanding, given that they should be taken into account for both perceived benefits and simplicity of usage. Thirdly, encourage students in higher education to use flipped classrooms by educating them about the various benefits of technology use and providing them with course materials or other learning objectives relevant to the sustainability of lengthy training. More study is required to determine how the intricacy of e-learning platforms and their connections to other educational standards, such as learning management systems (LMS), relate to higher education.

## Declarations

### Author contribution statement

Ibrahim AlYoussef: Conceived and designed the experiments; Performed the experiments; Analyzed and interpreted the data; Contributed reagents, materials, analysis tools or data; Wrote the paper.

### Funding statement

This work was supported by the Deanship of Scientific Research, Vice Presidency for Graduate Studies and Scientific Research, King Faisal University, Saudi Arabia [Grant No. 2115].

### Data availability statement

Data included in article/supp. material/referenced in article.

### Declaration of interest’s statement

The authors declare no conflict of interest.

### Additional information

Supplementary content related to this article has been published online at [URL].

## Appendix A Supplementary data

Questionnaire (Flipped) Questionnaire (Flipped)

Ethical Clearance (Flipped learning) Ethical Clearance (Flipped learning)
