# Supplementary material for: Acceptance of a flipped classroom to improve university students’ learning: An empirical study on the TAM model and the unified theory of acceptance and use of technology (UTAUT)
Source: Heliyon. 2022 Dec 22;8(12):e12529. doi: 10.1016/j.heliyon.2022.e12529 (PMC9816777; doi:10.1016/j.heliyon.2022.e12529)
Supplement: Questionnaire (Flipped) [file mmc1.docx]

**Questionnaire**

| **Peer influence (PI)** | | |
| --- | --- | --- |
| 1. | | My friends think that using flipped classroom is valuable for learning |
| 2. | | My friends’ opinions are important to me. |
| 3. | | If most of my friends started using flipped classroom for learning, it would encourage me to do the same. |
| 4. | | People who are important to me think that I should use flipped classroom |
| 5. | | People whose opinions that I value prefer that I use flipped classroom |
| **Relative advantage (ADV)** | | |
| 6. | | Improve the quality of my learning. |
| 7. | | Make it easier for my learning. |
| 8. | | Enhance my learning effectiveness |
| 9. | | Increase my productivity. |
| 10. | | Using flipped classroom will help me to learn more about my study. |
| **Perceived anxiety(PA)** | | |
| 16. | Learning through a flipped classroom would make me very nervous | |
| 17. | I get a sinking feeling when I think of trying to use a flipped classroom as a learning tool | |
| 18. | Learning through flipped classroom make me feel uncomfortable | |
| 19. | Learning through flipped classroom make me feel uneasy and confused | |
| 20. | I feel apprehensive about using flipped classroom as a learning tool | |
| **Perceived enjoyment (PE)** | | |
| 21. | I think the flipped classroom approach is an attractive and innovative method of teaching | |
| 22. | The flipped classroom approach is a high efficacy and positive method in the educational field | |
| 23. | Learning at home and doing homework and discussion in the classroom is a pleasant method and an attractive recommendation for me | |
| 24. | The flipped classroom approach is an exciting teaching method | |
| 25. | The flipped classroom approach is an enjoyable method | |
| **performance expectancy (PEX)** | | |
| 26. | flipped classroom instruction would enable me to accomplish tasks related to my studies more quickly | |
| 27. | flipped classroom instruction would help me to improve my grades | |
| 28. | flipped classroom instruction would enable me to learn on my pace | |
| 29. | flipped classroom instruction would enable me to find related links to academic matters easily | |
| 30. | Flipped classroom is necessary for my studies. | |
| **Effort expectancy (EEX)** | | |
| 31 | I regard Web-based instruction as new and interesting like trying something new | |
| 32. | Interacting with Web-based instruction is clear | |
| 33. | Learning how to use Web-based instruction is  would be easy | |
| 34. | It is would be easy to use Web-based instruction | |
| 35. | Interacting with Web-based instruction is understandable | |
| **Facilitating conditions (FC)** | | |
| 36 | When I need help to use Flipped Classroom, specialized instruction is available to help me | |
| 37. | When I need help to use Flipped Classroom, a specific person is available to provide assistance. | |
| 38. | When I need help to use Flipped Classroom, guidance is available to me | |
| 39. | I have the technical knowledge and resources needed to use Flipped Classroom | |
| 40. | The teaching staff has designed interesting questions and activities for Flipped Classroom | |
| **Perceived usefulness (PU)** | | |
| 41. | Using Flipped Classroom will improve my learning. | |
| 42. | Using Flipped Classroom will increase my productivity | |
| 43. | Using Flipped Classroom will enhance my effectiveness | |
| 44. | I find Flipped Classroom a useful tool in my learning. | |
| 45. | I believe using the flipped classroom approach accelerates the learning process | |
| **Behavioural intention to use Flipped Classroom(BIFC)** | | |
| 46. | I intend to participate in the flipped classroom in the future | |
| 47. | I predict that I can make important advancements in learning using the flipped classroom | |
| 48. | Using the flipped classroom is one of my plans in the future | |
| 49. | Using the flipped classroom is a useful and effective method, and this encourages me to attend these classes | |
| 50. | I would like to continue using flipped classroom instruction in my present and future teaching initiatives | |
| **Adoption of Flipped Classroom (AFC)** | | |
| 51. | I currently use flipped classroom in my study frequently. | |
| 52. | I use flipped classroom to improve my study skills. | |
| 53. | I feel that flipped classroom tools would be easy to use in my study. | |
| 54. | I use flipped classroom for getting resources from my lecturers and supervisors. | |
| 55. | I use flipped classroom for sharing resources with peers and group members. | |
