# Supplementary material for: Acceptance of a flipped classroom to improve university students’ learning: An empirical study on the TAM model and the unified theory of acceptance and use of technology (UTAUT)
Source: Heliyon. 2022 Dec 22;8(12):e12529. doi: 10.1016/j.heliyon.2022.e12529 (PMC9816777; doi:10.1016/j.heliyon.2022.e12529)
Supplement: Ethical Clearance (Flipped learning) [file mmc2.pdf]

Deanship of Scientific Research

عمادة البحث العلمي

موافقة أخلاقية

Ethical Clearance

Ref. No.	KFU-REC-2022- AUG – ETHICS116	الرقم المرجعي
Project Title	Acceptance of a Flipped Classroom to Improve University Student for Learning: A Study among Saudi Arabia University Students	اسم المشروع البحثي
Applicant	Ibrahim Youssef Alyoussef	اسم صاحب الطلب
Phone	00966558028222	الهاتف
Email	ialyoussef@kfu.edu.sa	البريد الإلكتروني
Granting Institution	جامعة الملك فيصل<br>King Faisal University	المؤسسة المانحة
Approval Date	07/06/2022	تاريخ الموافقة
Approval Validity	24 Months	صلاحية الموافقة

تفيد لجنة أخلاقيات البحث العلمي بجامعة الملك فيصل بأنه تم منح المشروع البحثي الموضح عاليه موافقة أخلاقيات البحث العلمي، وذلك بناء على فحص للجانب الأخلاقي من المشروع وفقاً للبيانات المزودة من قبل صاحب الطلب. قد تخضع المشاريع البحثية للمتابعة الميدانية أو أي شكل آخر من أشكال المتابعة الدقيقة من قبل اللجنة في أي وقت. قد تطلب اللجنة تقريرًا منتظمًا عن تقدم المشروع لضمان التزام الباحثين بأعلى المعايير الأخلاقية. الباحثون مسؤولون عن تخزين وحفظ وتأمين البيانات الناجمة عن المشاريع. يجب على الباحثين إبلاغ اللجنة على الفور بأي تعديلات جوهرية على المشروع أو قضايا أخلاقية مستجدة وذلك من خلال البريد الإلكتروني (ialjreesh@kfu.edu.sa) أو الهاتف (0096615899773).

Having reviewed the details submitted by the applicant regarding the abovenamed research project, the Research Ethics Committee at King Faisal University grants its ethical approval to the protocol. Projects may be subject to an audit or any other form of monitoring by the committee at any time. The committee may request a regular report on the progress of the project to ensure that researchers are committed to the highest ethical standards. Researchers are held accountable for the storage, retention and security of original data obtained from projects. Any substantial alterations to the project or emerging events or matters that may affect the ethical acceptability of the project must be reported immediately to the committee via email (ialjreesh@kfu.edu.sa) or phone (0096615899773).

The Chair of the Research Ethics Committee	أ.د. عبدالرحمن الليلي<br>Prof. Abdulrahman Al Lily	رئيس لجنة أخلاقيات البحث العلمي
Date	8/23/2022	التاريخ

*هذه الموافقة مشروطة بمصادقة معالي رئيس الجامعة على المحضر
